# P02.149. Integrative integrated migraine care: preliminary evaluation

**DOI:** 10.1186/1472-6882-12-S1-P205

**Published:** 2012-06-12

**Authors:** R Lauche, H Cramer, J Vasmer, A Paul, G Dobos, T Rampp

**Affiliations:** 1 University of Duisburg, Essen, Germany

## Purpose

Chronic migraine is one of the most common neurological disorders. Different complementary therapies are available for the treatment of these patients. The Department of Integrative Medicine at the University of Duisburg-Essen offers an integrative integrated migraine care model (IIMC) which is reimbursed by one of the biggest German statutory health insurance companies. Treatment options include naturopathic treatments, Traditional Chinese Medicine including acupuncture and herbs, Mind/Body interventions, and self-help strategies in an outpatient or inpatient setting as well as in a day clinic. For a preliminary evaluation of the effectiveness of the IIMC, we investigated the clinical outcomes of 28 patients who had completed the treatment by December 2010.

## Methods

All patients who had completed InVers before December 2010 were asked to fill in a questionnaire about their migraine before, directly after (post1), and 6 months after IIMC (post2). Further data were gained from medical records. Outcomes included frequency and intensity of migraine attacks, medication use and a 5-point Likert global improvement scale.

## Results

Twenty-eight patients returned completed questionnaires. Their mean age was 44.9±10.1 years. Patients suffered from 11.5±7.3 attacks a month before treatment; they were reduced to 5.8±5.0 (post1) and 5.6±4.0 (post2) (p=0.002). Migraine pain intensity dropped from 7.5±1.2 to 5.7±2.2 (post1, post2) (p<0.001). Days under medication were reduced more than 50% from 9.8±6.0 to 4.3±4.2 (post1) and 4.6±3.9 (post2) (p<0.001). Global improvement scale indicated that 17 out of 27 patients rated their health at least somewhat better than before IIMC.

## Conclusion

The integrative integrated migraine care model seems to be effective in reducing frequency and intensity of migraines and helps reducing migraine medication use. After this first evaluation, a larger prospective observational study is currently being conducted. This study also focuses on quality of life and changes in self-efficacy.

